# Developmental aspects of FXAND in a man with the *FMR1* premutation

**DOI:** 10.1002/mgg3.1050

**Published:** 2020-01-03

**Authors:** Ellery Santos, Chinelo Emeka‐Nwonovo, Jun Yi Wang, Andrea Schneider, Flora Tassone, Paul Hagerman, Randi Hagerman

**Affiliations:** ^1^ MIND Institute University of California Davis School of Medicine Sacramento CA USA; ^2^ Department of Pediatrics University of California Davis School of Medicine Sacramento CA USA; ^3^ Center for Mind and Brain University of California Davis Sacramento CA USA; ^4^ Department of Biochemistry and Molecular Medicine University of California Davis School of Medicine Sacramento CA USA

**Keywords:** ASD, *FMR1*, Fragile X, FXAND, Premutation

## Abstract

**Background:**

Fragile X mental retardation 1 (*FMR1*) premutation can cause developmental problems including autism spectrum disorder (ASD), social anxiety, depression, and attention deficit hyperactivity disorder (ADHD). These problems fall under an umbrella term of Fragile X‐associated Neuropsychiatric Disorders (FXAND) and is separate from Fragile X‐associated Tremor/Ataxia syndrome (FXTAS), a neurodegenerative disorder.

**Methods/Clinical Case:**

A 26‐year‐old Caucasian male with the Fragile X premutation who presented with multiple behavior and emotional problems including depression and anxiety at 10 years of age. He was evaluated at 13, 18, and 26 years old with age‐appropriate cognitive assessments, psychiatric evaluations, and an MRI of the brain.

**Results:**

The Autism Diagnostic Observation Scale (ADOS) was done at 13 years old and showed the patient has autism spectrum disorder (ASD). An evaluation at 18 years old showed a full‐scale IQ of 64. A Kiddie Schedule for Affective Disorders and Schizophrenia (K‐SADS) performed at 26 years old confirmed the previous impression of social anxiety disorder, agoraphobia disorder, and selective mutism. His MRI acquired at 26 years old showed enlarged ventricles, increased frontal subarachnoid spaces, and hypergyrification.

**Conclusion:**

This is an exemplary case of an *FMR1* premutation carrier with significant psychiatric and cognitive issues that demonstrates Fragile X‐associated Neuropsychiatric Disorders (FXAND) as separate from the other well‐known premutation disorders.

## Graphical abstract

*FMR1* premutation carriers develop neuropsychiatric problems that present in childhood and persists into adulthood. We review and characterize the features of Fragile X‐associated Neuropsychiatric Disorders (FXAND) in an exemplary case.

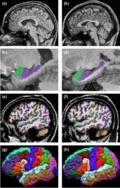

## 1 INTRODUCTION

Most individuals with the premutation (55 to 200 CGG repeats) in the Fragile X Mental Retardation 1 gene (*FMR1*, OMIM: 309,550) do not have intellectual disability in contrast to those with Fragile X Syndrome (FXS) that is caused by the full mutation (>200 CGG repeats) in *FMR1*. However, those with the premutation often have other problems including the Fragile X‐associated Primary Ovarian Insufficiency (FXPOI) before age 40, seen in approximately 20% of female carriers; and the Fragile X‐associated Tremor/Ataxia syndrome (FXTAS), a neurodegenerative disorder, seen in approximately 45% of male and 16% of female carriers with the premutation after age 50 (Hagerman & Hagerman, 2016). The cause of these clinical problems is related to the elevated *FMR1*‐mRNA that correlates positively with the increase in CGG repeat number in the premutation range. The elevated mRNA can be toxic to the cell and is associated with sequestration of vital proteins important for neuronal functions, mitochondrial dysfunction, calcium dysregulation, and chronic DNA damage repair (Hagerman & Hagerman, 2016).

The premutation can also cause developmental problems including social deficits, autism spectrum disorder (ASD), social anxiety, depression, obsessive compulsive disorder, and attention deficit hyperactivity disorder (ADHD) (Chonchaiya et al., 2012; Farzin et al., 2006). These problems in addition to adult disorders such as chronic fatigue, central pain syndrome, migraines, and autoimmune dysfunction fall under an umbrella term of Fragile X‐associated Neuropsychiatric Disorders (FXAND). At least one of these problems occurs in the majority of premutation carriers. Bailey, Raspa, Olmsted, and Holiday (2008) found developmental problems in about 30% of premutation carriers. These problems are related to mRNA toxicity and mitochondrial deficits documented in children with psychiatric problems and the premutation (Napoli et al., 2018; Song et al., 2016). Premutation neurons in culture have higher rates of spikes and they also die faster in culture compared to neurons without the premutation (Chen et al., 2009). In addition, GABA deficits have been documented in premutation carriers (Conde et al., 2013; D'Hulst et al., 2006).

The prevalence of the premutation is approximately 1 in 200 girls and 1 in 400 boys from newborn screening in the US (Tassone et al., 2012). However, in other countries the premutation prevalence may be much higher, such as Colombia, Israel and Mallorca (Saldarriaga et al., 2018). The prevalence of ASD in premutation males depends on whether they are the proband of the family who presents to a developmental center or whether they are identified by cascade testing (non‐proband) once another family member demonstrates an *FMR1* mutation. In the study by Chonchaiya et al. (2012), of 50 boys with the premutation, 68% of the probands had ASD whereas 28% of the non‐probands had ASD and none of the controls had ASD. Overall 14% of the boys with the premutation had seizures and the presence of seizures was highly correlated with ASD (Chonchaiya et al., 2012). Although most of the boys in these studies had a normal IQ, a subgroup had mild intellectual disability (Chonchaiya et al., 2012).

Here we report a patient with the premutation who has been followed clinically from childhood into adulthood to demonstrate the trajectory of FXAND symptoms over time.

## 2 CASE PRESENTATION

The patient is a 26‐year‐old Caucasian male with the Fragile X premutation documented via *FMR1* (GenBank: NG_007529.2) DNA testing who has been diagnosed with selective mutism, agoraphobia, and social anxiety disorder initially presented at 13 years of age. His CGG repeat size was 56, while his *FMR1* mRNA was 2.09 (± 0.12) times normal. He was born full‐term after an uneventful pregnancy. He did not require resuscitation and did well in the newborn period.

Early milestones were appropriate; he sat at 6 months, began crawling at 8 months and walking at 13 months. At 18 months he was saying 4 words but regressed and lost his ability to speak. During this time, he received speech and language therapy and by the age of 4 he began to speak with words again. By 6 years of age he was speaking spontaneously in sentences. Initially he was diagnosed with dyspraxia. However, he had a history of selective mutism particularly when seeing new people or in visiting physicians. He has several autistic features including poor eye contact, preservations, tantrums, and tactile defensiveness. He is also very sound sensitive and chews on his nails excessively. He had problems falling asleep at night which has been attributed to poor sleeping habits. He was late with toilet training for bowel movements until 8 years of age related to his anxiety. At 10 years of age he was diagnosed with multiple behavior and emotional problems including depression and anxiety, which is contributing to his selective mutism. At the time of the evaluation at 13 years of age he was started on fluoxetine initially, then later citalopram. Eventually he was started on sertraline and aripiprazole to improve his mood and anxiety.

His issues with depression, anxiety, and selective mutism have continued from childhood into adulthood. Even with language and cognitive therapy in conjunction with pharmacological interventions, his behavior showed minimal improvement.

The patient was seen in the MIND Institute, UC Davis Medical Center at age 13, 18, and 26 years old. He demonstrates poor eye contact, some sensory issues, shyness, and social anxiety during assessments. The Autism Diagnostic Observation Schedule (ADOS) model 3 was done at 13 years of age and he received a score of 12, which falls in the autism spectrum disorder range.

His Vineland Adaptive Behavior Scales (VABS) score at 13 years of age was 74, with communication at 77, daily living skills at 81 and socialization at 71. These test results confer an adaptive level that is moderately low. He was also assessed with the Wechsler Intelligence Scale for Children‐IV (WISC‐IV) at age 13, but because of his oppositional behavior, testing results were limited. His score included two subtests with estimated scores of 53 for perceptual reasoning index (PRI) and 56 for processing speed (PSI). The Wechsler Adult Intelligence Scale (WAIS‐IV) was performed at 18 years of age showing a full‐scale IQ of 64. Table 1 summarizes the cognitive assessment results.

**Table 1:** Comparing cognitive assessment from 2006 and 2011

Items	2006 WISC‐IV	2011 WAIS‐IV

Verbal Comprehension	N/A	63
Perceptual Reasoning	53	80
Working Memory	N/A	65
Processing Speed	56	66

Verbal IQ		62
Performance IQ		74
Full Scale IQ		64

Note

Results given in Standard Score (Mean 100, *SD* 15)

The psychiatric evaluation was performed at 26 years of age using Kiddie Schedule for Affective Disorders and Schizophrenia (K‐SADS). The K‐SADS confirmed the previous impression of social anxiety disorder and oppositional defiant disorder (subthreshold) in addition to agoraphobia disorder and selective mutism. He did not meet the diagnosis of Autism. Per mother, the patient is described as having anxiety issues in new situations, refusing to cooperate, obstinacy, anger issues, and “shutting down” when communicating with new people. His anxiety and depressed mood became apparent at around 10 years of age when he began 5th grade in a public school. At that time, he had become self‐destructive and would threaten self‐harm. His mood improved after starting a school that catered to children with special needs.

**Figure 1:**
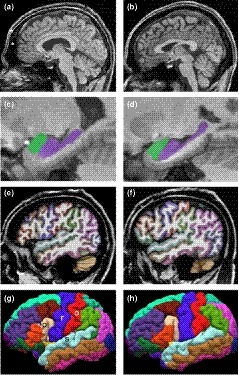
MRI findings. (a, c, e, g) The 26‐year‐old premutation carrier. (b, c, f, h) A 24‐year‐old male control. (a, b) A mid‐sagittal slice showing enlarged lateral ventricles and enlarged frontal subarachnoid spaces in the carrier (*). (c, d) Disproportionally enlarged hippocampal head the length of which is normally 35% of the whole length (Hackert et al., 2002).^23^ Hippocampus, purple; amygdala, lime. (e, f) A sagittal slice of the left hemisphere showing more complex folding in the carrier at the junction of the frontal, parietal, and temporal lobes. (g, h). A lateral view of the reconstructed cortex of the left hemisphere showing more complex folding in the pars opercularis (p, light brown, LGI = 4.49, *Z* = 2.38, 1.73%), precentral gyrus (r, purple, LGI = 3.79, *Z* = 4.32, 0.0008%), postcentral gyrus (o, red, LGI = 3.81, *Z* = 3.81, 0.007%), superior temporal gyrus (s, cyan, LGI = 4.32, *Z* = 2.58, 0.49%), and transverse temporal cortex (*, gray, LGI = 5.22, *Z* = 4.00, 0.0032%). Additional areas showing increased LGI are: left entorhinal cortex (LGI = 2.54, *Z* = 3.14, 0.085%), right postcentral gyrus (LGI = 3.72, *Z* = 2.28, *p* = .023), right superior temporal gyrus (LGI = 4.40, *Z* = 5.00, <0.0001%), and right transverse temporal gyrus (LGI = 5.07, *Z* = 2.66, 0.39%)

His magnetic resonance imaging (MRI) scans acquired at 26 years of age show enlarged lateral ventricles, increased frontal subarachnoid spaces, disproportionally large hippocampal head (bilateral), and hypergyrification (i.e., complex cortical folding) (Figure 1). To confirm these observations, *Z* scores of the volumes of the four ventricles and whole brain and local gyrification indices (LGI) of 68 cortical areas were computed relative to 8 age‐matched healthy males (age mean 25.9 years, *SD* 1.27 years, range 24–28 years). The volumetric data were obtained using Brain GPS (Mori et al., 2016; Tang et al., 2013; Wang et al., 2013) followed by machine‐learning based error correction and manual editing (Wang et al., 2011; Wang, Ngo, Hessl, Hagerman, & Rivera, 2016). LGI (Schaer et al., 2008) a measure of gyrification by the ratio of total surface area (both hidden within the sulci and exposed on the outer surface of the brain) and exposed surface area was quantified using Freesurfer (Dale, Fischl, & Sereno, 1999; Fischl, Sereno, & Dale, 1999). While the volumes of 3rd and 4th ventricles were in the normal range (3rd/4th Z = –0.91/0.45, 63.7%/65.3%), lateral ventricles were 2.2‐fold enlarged (35.2 ml, *Z* = 4.09, 0.0043%) and normalized whole brain volume was slightly reduced (1.51 L, *Z* = –1.90, 94.3%). In addition, he showed significantly increased LGI in the areas important for sensorimotor, auditory, language, and memory functions (*Z* = 2.28–5.00, 0.0001%–2.26%).

## 3 DISCUSSION

This case is an example of a premutation carrier with psychiatric and cognitive features that can be seen in premutation carriers with FXAND. These problems are significantly different from that of FXPOI and FXTAS, the other well‐known premutation disorders. The patient did not meet the criteria of FXTAS because he did not experience tremor/ataxia, nor did he have MRI findings found in FXTAS. This presentation is unusual for a carrier with only 56 repeats and *FMR1* mRNA at twice normal levels.

Extensive molecular and mitochondrial studies have already demonstrated an association with psychiatric disorders and premutation carrier status (Hagerman & Hagerman, 2016; Song et al., 2016) and that they can occur before neurological problems develop in FXTAS (Hall et al., 2016). The most common psychiatric problems seen in young boys with the premutation is anxiety (Cordeiro, Abucayan, Hagerman, Tassone, & Hessl, 2015). Cordeiro et al. (2015) studied 35 premutation carriers with the standardized Anxiety Disorders Interview Schedule for DSM‐IV (ADIS‐IV). They demonstrated that children and adult fragile X premutation carriers are at a higher risk for anxiety disorders, including Generalized Anxiety Disorder, Specific Phobia, Social Phobia and Obsessive‐Compulsive Disorder than the general population. This patient presented with psychiatric problems typical in young premutation carriers, particularly with boys having a higher incidence of ASD (Farzin et al., 2006; Seritan, A., Ortigas, M., Seritan, S., A. Bourgeois, J., & J. Hagerman, R., 2013; Chonchaiya et al., 2012). The rates of ASD range from 8% to 73% depending on whether they presented through cascade testing or as a proband for their family (Farzin et al., 2006; Chonchaiya et al., 2012).

The severity of the anxiety disorder has been shown to correlate with the presence of ASD in male carriers which is consistent with this case study (Cordeiro et al., 2015). Other environmental exposures or “background” genetic modifiers likely effect the presentation of clinical manifestations in carriers (Muzar, Adams, Schneider, Hagerman, & Lozano, 2014). Chonchaiya et al. (2012) demonstrated that seizures in males with the premutation are associated with a higher rate of ASD, although our patient did not have clinical seizures.

MRI findings found in this patient were consistent with his clinical symptoms and with previous documentation of structural changes that can begin in childhood of premutation carriers (Wang et al., 2017). The enlarged lateral ventricles and increased frontal subarachnoid spaces may be related to poor absorption of cerebrospinal fluid that would lead to fluid stagnation and sleep problems (Mortazavi et al., 2018; Ringstad et al., 2018). Disproportionally large hippocampal head and hypergyrification (especially in the primary sensorimotor and auditory cortices) may reflect defective embryonic neurodevelopment that have been shown in knock‐in mouse model of the premutation and may be associated with developmental delay, tactile defensiveness, sound sensitive, selective mutism, depression, and anxiety (Cunningham et al., 2010).

Molent et al. (2017) demonstrated increased hypergyrification and reduce cortical thickness is associated with generalized anxiety disorder (GAD) in the general population, suggesting a neurodevelopmental origin. This study is the first report of hypergyrification in a premutation carrier. In animal studies, we know that migration of neurons is impacted by the premutation (Cunningham et al., 2010). These MRI findings may prove useful in tracking GAD in premutation carriers as well as the changes in cerebral morphology as these patients age and begin to develop other neurological symptoms.

Premutation carriers have shown large phenotypic variability, ranging from FXTAS (Hagerman & Hagerman, 2013), psychiatric disorders (Seritan et al., 2013) to neurodevelopmental disorders (Farzin et al., 2006). This underlies the importance of identifying FXAND as a separate group of premutation disorders which require further treatment studies. Patients with this disorder suffer from significant psychiatric issues that can occur during childhood before any symptoms of FXTAS. It is hypothesized that the RNA toxicity and mitochondrial dysfunction that occurs in FXTAS may also be the pathogenesis of FXAND, but further studies are needed. It is also possible that this patient has additional genetic mutations that further led to his symptoms, but further studies of his genome are necessary to confirm this hypothesis.

## CONFLICT OF INTEREST STATEMENT

Randi Hagerman has received funding from Zynerba and Ovid for carrying out treatment studies in patients with fragile X syndrome. She has also consulted with Fulcrum, Ovid and Zynerba regarding treatment studies in individuals with fragile X syndrome. Dr. Tassone has received funding from Zynerba and Asuragen, Inc. The other authors declare no conflicts of interest.

## AUTHOR CONTRIBUTIONS

E.S., C.E., J.Y.W., and R.H. wrote the original draft along with reviewing and editing the manuscript. F.T., P.H., and R.H. conceived and presented the ideas. E.S. and C.E. contributed in data curation. J.Y.W., A.S., P.H., and R.H. collected the data, lead the investigation, and formal analysis. F.T., A.S., J.Y.W, and P.H. analyzed and interpreted the data. All contributing authors had final approval of the version to be published.

## Data Availability

The data that support the findings of this study are available on request from the corresponding author. The data are not publicly available due to privacy or ethical restrictions.
